# Effects of Berberis asiatica, Withania somnifera, and Their Combination on Body Weight in Streptozotocin-Nicotinamide-Induced Type 2 Diabetes in Wistar Rats

**DOI:** 10.7759/cureus.68295

**Published:** 2024-08-31

**Authors:** Devkumar D Tiwari, Vandana M Thorat, Prathamesh V Pakale, Sarika Patil

**Affiliations:** 1 Department of Pharmacology, Krishna Institute of Medical Sciences, Krishna Vishwa Vidyapeeth (Deemed to be University), Karad, IND

**Keywords:** ashwagandha (withania somnifera), herbal combination, synergestic effects, wistar rats, streptozotocin-nicotinamide, body weight, berberis asiatica, type-ii diabetes mellitus

## Abstract

Introduction

Type 2 diabetes mellitus (T2DM) is characterized by insulin resistance, impaired insulin secretion, and beta cell dysfunction, often leading to chronic hyperglycemia and associated complications. *Berberis asiatica* (*BA*) and *Withania somnifera* (*WS*) are ancient medicinal plants with a reputation for having potential therapeutic effects in diabetes management. The purpose of this study was to look into how body weight (BW) was affected in streptozotocin-nicotinamide (STZ-NIC) induced T2DM in Wistar rats by *BA*, *WS*, and their polyherbal combination (PHC).

Materials and methods

Seventy-eight Wistar rats of both sexes were divided into 13 groups, with six rats in each group, including normal and diabetic controls, and treated with varying doses of *BA*, *WS*, and PHC. The rats were under observation over the course of 35 days for any change in BW. The Organization for Economic Co-operation and Development (OECD) rules and guidelines were followed in the conduct of acute toxicity tests. One-way analysis of variance (ANOVA), followed by Tukey-Kramer post hoc tests, was used for statistical analysis.

Results

The findings indicated that the highest dose of *BA* (1000 mg/kg) significantly improved BW in diabetic rats, approaching that of the normal control group. The combination of *BA* and *WS* also demonstrated significant improvements in BW, suggesting a synergistic effect. The standard antidiabetic drugs, metformin and glimepiride, were effective in increasing BW in diabetic rats.

Conclusion

The study concludes that *BA*, *WS*, and their combination have a positive impact on BW management in T2DM rats, with the combination therapy showing enhanced effects. These findings support the potential utilization of these herbs in managing BW and other T2DM-associated metabolic disturbances and abnormalities.

## Introduction

Type 2 diabetes mellitus (T2DM) is characterized by insulin resistance, impaired insulin secretion, and beta cell dysfunction, leading to chronic hyperglycemia [1]. It often results in association with various metabolic disturbances and complications, such as cardiovascular diseases, nephropathy, and retinopathy [2]. One significant aspect of T2DM is its impact on body weight (BW) loss due to muscle wasting and fat breakdown [3]. While obesity is a well-known risk factor for T2DM, the disease itself can cause fluctuations in BW due to metabolic disturbances [4]. Conventionally used medicinal herbs, such as *Berberis asiatica* (*BA*) and *Withania somnifera* (*WS*), have been investigated previously for their potential therapeutic effects on diabetes [5]. This study aimed to investigate the effects of these plants, alone and in combination at a 1:1 ratio, on the BW of streptozotocin-nicotinamide (STZ-NIC) induced T2DM Wistar rats.

STZ-NIC-induced diabetic model

The STZ-NIC-induced diabetic model in Wistar rats is a well-established and widely used experimental model to study T2DM [6]. STZ selectively destroys insulin-producing beta cells in the pancreas [7], while NIC partially protects against STZ's cytotoxic effects, resulting in an animal model that mimics the insulin resistance and partial insulin deficiency seen in human T2DM [8]. This model is particularly useful for studying the metabolic consequences of diabetes, including changes in BW.

BA

A medicinal plant commonly known as Indian barberry (Daaru haldi) in India and (Chutro) in Nepal is a perennial shrub belonging to the family Berberidaceae. It is mostly available in Himalayan areas, which include portions of China, Nepal, Bhutan, and India [9]. It thrives at altitudes ranging from 800 to 4000 meters above sea level, particularly in temperate and sub-alpine zones [10]. It has a rich composition of bioactive compounds, notably alkaloids such as berberine, palmatine, and jatrorrhizine. Berberine, in particular, is the most studied compound due to its broad spectrum of pharmacological activities. Other significant constituents include flavonoids, tannins, phenolic acids, and essential oils [11]. *BA* has been employed to treat various diseases and illnesses in the traditional medicine systems of India (Ayurveda, Siddha, Unani) and China (Traditional Chinese Medicine) for centuries because of its therapeutic properties, such as antimicrobial, anti-inflammatory, antioxidant, hepatoprotective, and hypoglycemic activities. *BA* stands out as an essential orthodox medicinal herb with diverse pharmacological benefits. The wealth of bioactive compounds it harbors, particularly berberine, underscores its potential in developing pharmaceutical agents for a wide range of illnesses and health conditions [12].

WA

Traditional medicinal herb, commonly known as Ashwagandha or Winter cherry, is a prominent herb in traditional Ayurvedic medicine. It comes from the Solanaceae family and is additionally referred to as "Indian ginseng" because of its rejuvenating properties. It is a tiny shrub with red berries and yellow blooms, native to India, the Middle East, and Africa [13]. It has been used for centuries in Ayurvedic medicine as a rasayana (rejuvenative) and adaptogen [14]. It is traditionally believed to enhance physical and mental health, improve vitality, and increase longevity. The plant’s root is primarily utilized for medicinal purposes, although the leaves, seeds, and fruits are also used in some treatments [15]. *WS* is attributed to its rich composition of bioactive compounds; key constituents include withanolides, withaferins, alkaloids, and sitoindosides. Withanolides, particularly withaferin A, are steroidal lactones that exhibit a wide range of biological activities, including anti-inflammatory, anti-cancer, anti-stress, and neuroprotective effects [16]. Many of the traditional applications of Ashwagandha have been confirmed by the latest scientific research, demonstrating its potential in modern medicine. Some of the notable pharmacological effects include adaptogenic properties [14], antioxidant activities [17], and muscle mass preservation [18]. The therapeutic applications of *WS* are diverse. It is commonly used to improve sleep, boost the immune system, enhance physical performance, and manage conditions like arthritis and diabetes. Ongoing research continues to explore its efficacy and safety in various health conditions.

Rationale for combination therapy

The combination of *BA* and *WS* in the ratio of 1:1 holds promise for a synergistic approach to T2DM management [5]. Combining their complementary mechanisms of action could provide enhanced therapeutic benefits, such as improved glycemic control [19], better BW management [20], and comprehensive metabolic benefits [21].

## Materials and methods

Experimental animal

A total of 78 Wistar rats of both sexes, aged five to six weeks (150-250 g), were obtained from the Central Animal House of the Krishna Institute of Medical Sciences, Krishna Vishwa Vidyapeeth (KVV), Karad, India. The rats were housed individually at 27-37°C and 50% humidity on a 12-hour light-dark cycle (lights on at 6:00 am) and had access to a standard diet and filtered drinking water ad libitum. The rats were acclimatized for seven days before the initiation of the experiment. The experimental protocol was approved by the Institutional Animal Ethics Committee of KVV (Deemed to be University), Reg. no. 255/PO/REBi/S/2000/CPCSEA (IAEC approval no. IAEC/KIMS/2021/16).

All the experiments were conducted according to the guidelines of the Committee for Control and Supervision of Experiments on Animals (CCSEA) in the Central Animal House, KVV. Metformin (MET), glimepiride (GLI), *BA*, and *WS* were dissolved in distilled water and administered orally. Standard antidiabetic drugs, herbal drugs, and herbal combination doses were prepared immediately before use and given orally from the seventh day daily until the study’s completion (35th day). Doses of the drugs and herbal combinations were selected based on previous studies conducted in our laboratory and from the literature.

Acute oral toxicity study

The acute oral toxicity study was successfully carried out in adult Wistar rats using the “limit dose” method of the Organization for Economic Co-operation and Development (OECD) guideline no. 240 [22]. A test procedure with a starting dose of 2000 mg/kg BW was adopted. The animals were fasted overnight, and the next day, extracts of the plants *BA* and *WS* were administered orally at a dosage of 2000 mg/kg BW. The animals were monitored continuously for three hours for general psychological, behavioral, neural, and autonomic characteristics, then every 30 minutes for the following three hours, and finally for mortality from 24 hours to 14 days.

Limit test

As per the OECD guidelines, it was ensured that the total dose of the polyherbal combination (PHC) did not exceed 2000 mg/kg BW, which is considered the upper limit dose for assessing acute toxicity. The second animal received the exact same dose as the initial one, and both survived after being given the upper limit dose. A total of three animals were administered the limiting dose, and as no deaths occurred, three additional animals of the other sex were tested at the same dose level. As there was no lethality, the test was terminated.

Table 1 consists of all 13 groups of rats, along with their specific drugs, dosage forms, and routes of administration. Each group consists of six animals.

**Table 1:** Experimental groups and treatment protocol NC: Normal control; DC: Diabetic control; *BA*: *Berberis asiatica*; *WS*: *Withania somnifera*; PHC: Polyherbal combination; MET: Metformin; GLI: Glimepiride

Group no.	Group name	Extract/drugs	Dose and route (orally) (mg/kg)
1	NC	Distilled water	10 mL/kg
2	DC	Distilled water	10 mL/kg
3	*BA* 250	Dried ethanolic root extract of *BA*	250
4	*BA* 500	Dried ethanolic root extract of *BA*	500
5	*BA* 1000	Dried ethanolic root extract of *BA*	1000
6	*WS* 250	Dried ethanolic root extract of *WS*	250
7	*WS* 500	Dried ethanolic root extract of *WS*	500
8	*WS* 1000	Dried ethanolic root extract of *WS*	1000
9	PHC 250	Dried ethanolic root extract of *BA* + *WS*	125 + 125
10	PHC 500	Dried ethanolic root extract of *BA* + *WS*	250 + 250
11	PHC 1000	Dried ethanolic root extract of *BA* + *WS*	500 + 500
12	MET	Metformin (standard)	250
13	GLI	Glimepiride (standard)	10

Drugs

The dried ethanolic root extract of *BA* and *WS* was procured from Natucare India Pvt. Ltd., Mumbai, India, and Bhagwati Herbal and Healthcare Pvt. Ltd., Vapi, India, in pure powder form. STZ and NIC were obtained from Sisco Research Laboratories Pvt. Ltd., Mumbai, India. MET and GLI were gathered from Smruthi Organic Ltd., Solapur, India, in pure powder form.

Induction of T2DM

T2DM was induced by injecting STZ (65 mg/kg BW) intraperitoneally (i.p.) in physiological saline, 15 minutes after NIC (110 mg/kg BW) administration i.p. [23]. The rats were kept and monitored for seven days after the injection of STZ-NIC. Random blood glucose (RBG) levels were examined using the tail vein prick method with a standard portable digital glucometer (BeatO Curv). The rats with RBG levels greater than 250 mg/dL were included in this investigation and were determined to be diabetic after the seventh day [24]. Accurate BW of all rats in the various experimental groups was measured in the experimental room of the Central Animal House, KVV Karad, using a transparent rat restrainer and a digital weighing balance, from day 1 to the end of the study on day 35, at weekly intervals.

Statistical analysis

The data were analyzed using a post hoc test after a one-way analysis of variance (ANOVA). The Tukey-Kramer test (post hoc test) was utilized to determine which group differences are statistically significant. Values of BW for all the groups are summarized as mean ± standard deviation. All p-value differences were considered statistically significant for p ≤ 0.05. All statistical analyses were performed with IBM SPSS Statistics for Windows, Version 18 (Released 2009; IBM Corp., Armonk, NY, USA).

## Results

Repeated ANOVA of *BA*

At day 0, there were no significant differences between the groups (NC, DC, *BA* 250, *BA* 500, *BA* 1000, MET, GLI), as the p-value was 0.9991, indicating no statistical significance. On day 7, the *BA* groups showed a significant difference in BW compared to the DC group but were similar to each other. BW decreased significantly in the DC group (p < 0.0001). On day 14, *BA* 1000 showed an increase in BW, which was significantly higher than in the DC group but not significantly different from the NC group. On day 21, *BA* 1000 continued to show improvement, with significant increases in BW compared to the DC group (p < 0.0001). On days 28 and 35, *BA* 1000 showed the most significant improvements in BW, nearing the weights of the NC group, and statistically significant differences from the DC group were observed (p < 0.0001). The repeated measures ANOVA also indicated significant differences over time (p < 0.0001).

Ordinary ANOVA of *BA*

**Figure 1:**
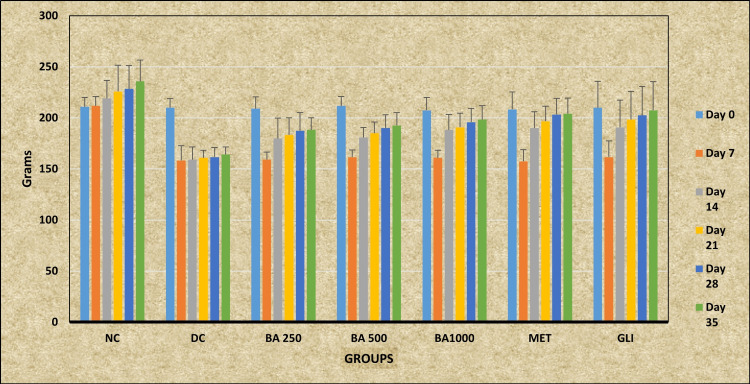
Effect of Berberis asiatica on body weight of rats induced with experimental diabetes NC: Normal control; DC: Diabetic control; MET: Metformin; GLI: Glimepiride; *BA*: *Berberis asiatica*

Followed by post hoc analysis, Tukey-Kramer multiple comparison tests revealed statistically significant greater BW in the study group NC at all the time intervals - day 7, day 14, day 21, day 28, and day 35 - except day 0 (p > 0.05) when compared with the DC group (p < 0.001). The *BA* 1000 group showed statistically significantly higher BW at day 28 and day 35 compared with the DC group (p < 0.05). Post hoc analysis also revealed that the MET and GLI groups showed statistically significant increased BW on day 21 (p < 0.05), day 28, and day 35 (p < 0.01) compared with the DC group. No statistically significant changes were observed in the *BA* 250, *BA* 500, and *BA* 1000 groups when compared with the NC group (p > 0.05) (Table 2; Figure 1).

**Table 2:** Effect of Berberis asiatica on body weight in diabetic rats a: DC differs significantly from *BA* 250, *BA* 500, *BA* 1000, MET, and GLI group; 1: p < 0.05; b: Day 0 differs significantly from day 7, day 14, day 21, day 28, and day 35; 2: p < 0.01; c: Day 7 differs significantly from day 0, day 14, day 21, day 28, day 35; 3: p < 0.001 F- and p-values presented in the column are from the ordinary ANOVA and Tukey-Kramer multiple comparison test. F and p-values presented in the row are from the repeated ANOVA and Tukey-Kramer multiple comparison test. NC: Normal control; DC: Diabetic control; *BA*: *Berberis asiatica*; MET: Metformin; GLI: Glimepiride; P: Probability; F: F-statistics; ANOVA: Analysis of variance

Groups		NC	DC	*BA* 250	*BA* 500	*BA* 1000	MET	GLI	Ordinary ANOVA	
Day									F-value	p-value
0		210.83 ± 9.17	210^c3 ^± 8.94	209^c3 ^± 11.58	211.67^c3 ^± 9.31	207.5^c3^ ± 12.55	208.33^c3 ^± 16.93	210^c3^ ± 25.88	0.05744	0.9991
7		211.67^a3^ ± 9.31	158.33^b3 ^± 14.38	159.17^b3^ ± 7.36	161.67^b3^ ± 6.83	160.83^b3^ ± 7.36	157.5^b3^ ± 11.29	161.67^b3 ^± 15.71	19.713	p < 0.0001
14		219.17^a3^ ± 17.44	159.17^b3^ ± 12.42	180^b3c2^ ± 19.89	180.83^b3c3 ^± 9.7	188.33^b3c3^ ± 15.06	190^b3c3^ ± 16.13	190.83^b2c3^ ± 26.46	6.513	p < 0.0001
21		225.83^a3^^b1 ^± 25.77	160.83^b3^ ± 7.36	183.33^b3c3^ ± 16.63	185^b3c3^ ± 10.95	190.83^b3c3^ ± 13.57	196.67^a1^^b2c3^ ± 14.72	198.33^a1^^c3^ ± 27.69	7.06	p < 0.0001
28		228.33^a3^^b1c1^ ± 22.95	161.67^b3^ ± 9.31	187.5^b2^^c3^ ± 17.54	190^b3c3^ ± 13.04	195.83^a1^^b3c3^ ± 13.57	203.33^a2^^c3^ ± 15.71	202.5^a1^^c3^ ± 28.06	7.358	p < 0.0001
35		235.83^a3^^b2c3^ ± 20.84	164.17^b3^ ± 7.36	188.33^b2c3^ ± 11.69	192.5^b3c3^ ± 12.55	198.33^a1^^b2c3^ ± 13.66	204.17^a1^^c3^ ± 15.30	207.5^a2^^c3^ ± 28.06	9.932	p < 0.0001
Repeated ANOVA	F-value	25.096	16.413	13.369	12.711	60.735	47.756	54.08	-	
	p-value	p < 0.0001	p < 0.0001	p < 0.0001	p < 0.0001	p < 0.0001	p < 0.0001	p < 0.0001		

Repeated ANOVA of *WS*

At day 0, there were no significant differences between the groups, similar to the *BA* group. On day 7, *WS* 1000 showed significant differences in BW compared to DC, similar to the trends observed with *BA*. On day 14, *WS* 1000 showed a substantial increase, aligning closer to NC group weights. On days 21 and 28, the trends continued, with *WS* 1000 maintaining significant differences from DC and being closer to NC. On day 35, *WS* 1000 showed significant improvement in BW, with statistically significant differences from DC (p < 0.0001). Significant differences were observed with p-values less than 0.0001 for all days. The DC group consistently showed lower BWs compared to the treatment groups.

Ordinary ANOVA of *WS*

Followed by post hoc analysis, the Tukey-Kramer multiple comparison tests revealed statistically significant higher BW in the study group NC at all the time intervals - day 7, day 14, day 21, day 28, and day 35 - except on day 0 (p > 0.05) when compared with the DC group (p < 0.001). The NC group also showed statistically significant higher BW on day 7 compared with the MET and GLI groups (p < 0.001).

**Figure 2:**
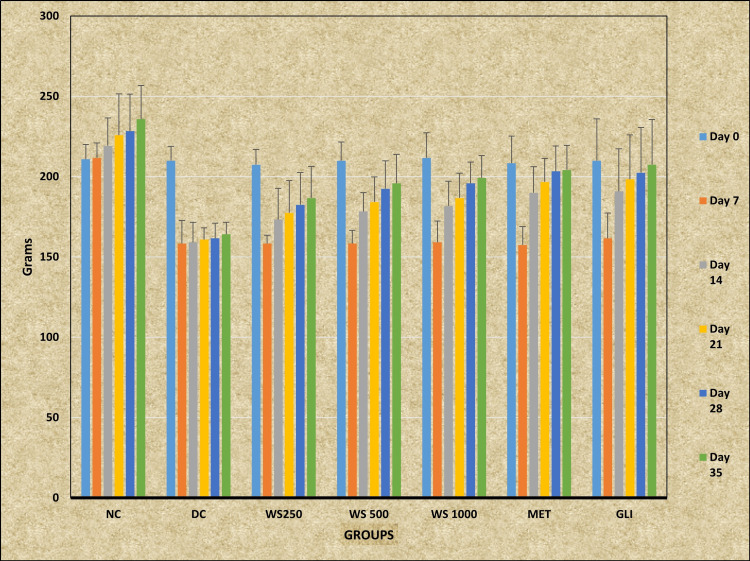
Effect of Withania somnifera on body weight of rats induced with experimental diabetes NC: Normal control; DC: Diabetic control; MET: Metformin; GLI: Glimepiride; *WS*: *Withania somnifera*

Analysis revealed that the *WS* 1000 group showed statistically significant higher BW on day 35 compared with the DC group (p < 0.05). Post hoc analysis also revealed that the MET group showed statistically significant increased BW on day 21 (p < 0.05), day 28 (p < 0.01), and day 35 (p < 0.05) compared with the DC group. The GLI group also showed a statistically significant rise in BW on day 21, day 28 (p < 0.05), and day 35 (p < 0.01) compared with the DC group. No statistically significant changes were observed in the *WS* 250, *WS* 500, and *WS* 1000 groups when compared with the NC group (p > 0.05) (Table 3; Figure 2).

**Table 3:** Effect of Withania somnifera on body weight in diabetic rats a: DC differs significantly from *WS* 250, *WS* 500, *WS* 1000, MET, and GLI group; 1: p < 0.05; b: Day 0 differs significantly from day 7, day 14, day 21, day 28, and day 35; 2: p < 0.01; c: Day 7 differs significantly from day 0, day 14, day 21, day 28, day 35; 3: p < 0.001 F- and p-values presented in the column are from the ordinary ANOVA and Tukey-Kramer multiple comparison test. F- and p-values presented in the row are from the repeated ANOVA and Tukey-Kramer multiple comparison test. NC: Normal control; DC: Diabetic control; MET: Metformin; GLI: Glimepiride; P: Probability; F: F-statistics; ANOVA: Analysis of variance; *WS*: *Withania somnifera*

Groups		NC	DC	*WS* 250	*WS* 500	*WS* 1000	MET	GLI	Ordinary ANOVA	
Day									F-value	p-value
0		210.83 ± 9.17	210^c3^ ± 8.94	207.5^c3^ ± 9.35	210 ^c3^ ± 11.58	211.67 ^c3^ ± 15.71	208.33^c3^ ± 16.93	210^c3^ ± 25.88	0.05768	0.9991
7		211.67^a3^ ± 9.31	158.33^b3^ ± 14.38	158.33^b3^ ± 5.16	158.33^b3^ ± 8.16	159.17^b3^ ± 13.20	157.5^b3^ ± 11.29	161.67^b3^ ± 15.71	17.955	p < 0.0001
14		219.17^a3^ ± 17.44	159.17 ^b3^ ± 12.42	173.33^b3^ ± 19.41	178.33^b3c2^ ± 11.69	181.67^b3c3^ ± 15.38	190^b3c3^ ± 16.13	190.83^b2c3^ ± 26.46	6.698	p < 0.0001
21		225.83^a3^^b1^ ± 25.77	160.83^b3^ ± 7.36	177.5 ^b3c2^ ± 19.94	184.17^b3c3^ ± 15.63	186.67^b3c3^ ± 15.38	196.67^a1^^b2c3^± 14.72	198.33^a1^^c3^ ± 27.69	6.647	p < 0.0001
28		228.33^a3^^b1c1^ ± 22.95	161.67^b3^ ± 9.31	182.5^b3c3^ ± 19.94	192.5^b2c3^ ± 17.25	195.83^b3c3^ ± 13.20	203.33^a2^^c3^ ± 15.71	202.5^a1^^c3^ ± 28.06	6.983	p < 0.0001
35		235.83^a3^^b2c3^ ± 20.84	164.17^b3^ ± 7.36	186.67^b2c3^ ± 19.66	195.83^b1c3^ ± 18.01	199.17^a1^^b2c3^ ± 13.93	204.17^a1^^c3^ ± 15.30	207.5^a2^^c3^ ± 28.06	8.206	p < 0.0001
Repeated ANOVA	F-value	25.096	16.413	17.391	14.62	41.142	47.756	54.08	-	
	p-value	p < 0.0001	p < 0.0001	p < 0.0001	p < 0.0001	p < 0.0001	p < 0.0001	p < 0.0001		

Repeated ANOVA of PHC

At day 0, there were no significant differences between the groups. On day 7, the PHC groups showed significant differences compared to DC, with PHC 1000 showing the most considerable improvement. On day 14, PHC 1000 showed increased BW, which was significantly different from DC. On days 21 and 28, PHC 1000 maintained the trend of significant improvement, nearing the weights of the NC group. On day 35, PHC 1000 had the highest improvement in BW among the PHC groups, being significantly different from DC (p < 0.0001). Significant differences were observed with p-values less than 0.0001 for all days. The DC group showed lower BW compared to the treatment groups.

Ordinary ANOVA of PHC

**Figure 3:**
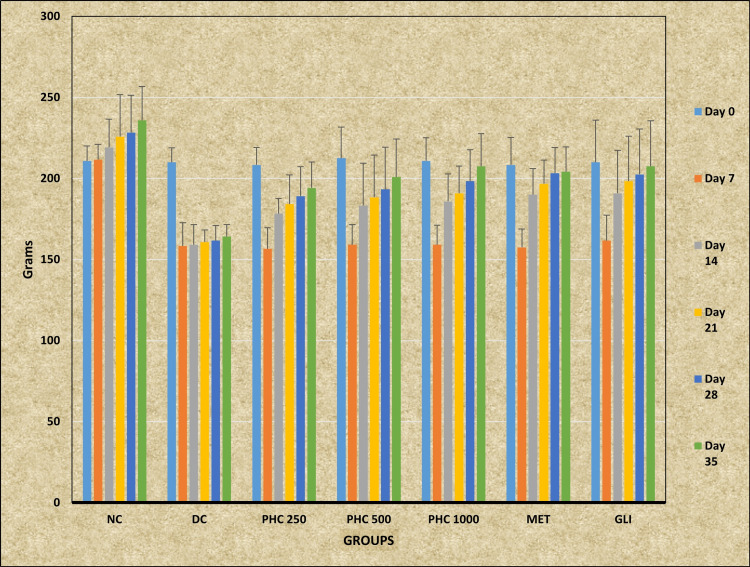
Effect of polyherbal combination on body weight of rats induced with experimental diabetes NC: Normal control; DC: Diabetic control; PHC: Polyherbal combination; MET: Metformin; GLI: Glimepiride

Followed by post hoc analysis, Tukey-Kramer multiple comparison tests revealed statistically significant higher BW in the study group NC at all the time intervals - day 7, day 14, day 21, day 28, and day 35 - except day 0 (p > 0.05) when compared with the DC group (p < 0.001). A statistically significant rise in BW was observed in the PHC 500 group (p < 0.05) and the PHC 1000 group (p < 0.01) on day 35 when compared with the DC group. The MET group showed statistically significantly higher BW on day 28 and day 35 in comparison with the DC group (p < 0.05). Post hoc analysis also revealed that the GLI group showed statistically significant increased BW on day 21, day 28 (p < 0.05), and day 35 (p < 0.01) when compared with the DC group (Table 4; Figure 3).

**Table 4:** Effect of polyherbal combination on body weight in diabetic rats a: DC differs significantly from PHC 250, PHC 500, PHC 1000, MET, and GLI group; 1: p < 0.05; b: Day 0 differs significantly from day 7, day 14, day 21, day 28, and day 35; 2: p < 0.01; c: Day 7 differs significantly from day 0, day 14, day 21, day 28, day 35; 3: p < 0.001 F- and p-values presented in the column are from the ordinary ANOVA and Tukey-Kramer multiple comparison test. F- and p-values presented in the row are from the repeated ANOVA and Tukey-Kramer multiple comparison test. NC: Normal control; DC: Diabetic control; MET: Metformin; GLI: Glimepiride; P: Probability; F: F-statistics; ANOVA: Analysis of variance; PHC: Polyherbal combination

Groups		NC	DC	PHC 250	PHC 500	PHC 1000	MET	GLI	Ordinary ANOVA	
Day									F-value	p-value
0		210.83 ± 9.17	210^c3^ ± 8.94	208.33^c3^ ± 10.80	212.5^c3^ ± 19.17	210.83^c3^ ± 14.29	208.33^c3^ ± 16.93	210^c3^ ± 25.88	0.05067	0.9994
7		211.67^a3^ ± 9.31	158.33^b3^ ± 14.38	156.67^b3^ ± 12.91	159.17^b3^ ± 12.42	159.17^b3^ ± 12.01	157.5 ^b3^ ± 11.29	161.67^b3^ ± 15.71	14.928	p < 0.0001
14		219.17^a3^ ± 17.44	159.17^b3^ ± 12.42	178.33^c3b3^ ± 9.31	183.33^b3c3^ ± 26.01	185.83^b3c3^ ± 16.86	190^b3c3^ ± 16.13	190.83^b2c3^ ± 26.46	5.439	0.0005
21		225.83^a3^^b1^ ± 25.77	160.83^b3^ ± 7.36	184.17^c3b3^ ± 18	188.33^b3c3^ ± 26.01	190.83^b3c3^ ± 16.86	196.67^a1^^b2c3^± 14.72	198.33^a1^^c3^ ± 27.69	5.283	0.0006
28		228.33^a3^^b1c1^ ± 22.95	161.67^b3^ ± 9.31	189.17^c3b3^ ± 18	193.33^b2c3^ ± 26.01	198.33^b1c3^ ± 19.41	203.33^a2^^c3^ ± 15.71	202.5^a1^^c3^ ± 28.06	5.499	0.0004
35		235.83^a3^^b2c3^ ± 20.84	164.17^b3^ ± 7.36	194.17^c3b1^ ± 15.94	200.83^c3^^a1^ ± 23.54	207.5^a2^^c3^ ± 20.19	204.17^a1^^c3^ ± 15.30	207.5^a2^^c3^ ± 28.06	6.944	p < 0.0001
Repeated ANOVA	F-value	25.096	16.413	21.495	48.016	46.153	47.756	54.08	-	
	p-value	p < 0.0001	p < 0.0001	p < 0.0001	p < 0.0001	p < 0.0001	p < 0.0001	p < 0.0001		

## Discussion

The management of T2DM often requires interventions that address hyperglycemia and associated complications, including BW regulation. STZ-NIC-induced T2DM in Wistar rats is a well-established model for studying T2DM and its related metabolic dysfunctions. This study evaluated the effects of *BA*, *WS*, and PHC on BW in STZ-NIC-induced T2DM Wistar rats in various groups, i.e., NC, DC, *BA* 250, *BA* 500, *BA* 1000, *WS* 250, *WS* 500, *WS* 1000, PHC 250, PHC 500, PHC 1000, and standard control groups MET and GLI. BW management is critical in DM, as weight loss is a common symptom due to muscle wasting and reduced adipose tissue caused by insulin deficiency, hyperglycemia-induced catabolism, and its effects on metabolism [25]. BW was measured over 35 days at seven-day intervals. On day 0, there were no significant differences in BW across all groups, indicating a uniform baseline before the treatments began. This is supported by the p-values from repeated measures ANOVA (p > 0.9991).

BA

In our study, we found that there were no differences between the groups (NC, DC, *BA* 250, *BA* 500, *BA* 1000, MET, GLI) at the start of the experiment (day 0). As we continued the study, we observed noticeable differences for all days until the end of the study (day 35). From day 7 onwards, the DC group showed a sharp decrease in BW compared to all other groups.

WS

*WS* showed no differences between the groups, similar to *BA*, at the start of the study (day 0). A similar pattern in differences was observed for all days and at all intervals for *WS* compared with *BA*. The DC group consistently showed lower BWs compared to the treatment groups, such as *BA*. We also observed a more prominent effect of *BA* at higher doses in managing decreased BW. After intervention with *WS* and *BA* at different dosages, the study showed successful inhibition of weight loss induced by diabetes in Wistar rats.

Standard drugs

Both groups, MET and GLI, showed significant improvements in BW compared to the DC group across all time points, from day 7 to day 35. On day 35, the MET and GLI groups reached BW similar to that of the PHC 1000 group.

PHC

On day 0, at the start of the study, no significant differences between the groups were observed, similar to *BA* and *WS*. From the second week of the study (day 7), differences were observed for all subsequent days. The DC group consistently showed lower BW compared to the treatment groups until the end of the study (day 35). PHC of *BA* and *WS* in a 1:1 ratio demonstrated a combined effect in regaining weight, surpassing the individual effects of *BA* and *WS* at all doses and time intervals, reflecting the superiority of PHC over individual treatments. A higher dose of PHC (1000 mg/kg BW) appeared to be a more potent and promising factor for inhibiting weight loss and promoting weight gain in diabetes-induced Wistar rats.

Mechanistic insight

BA

*BA* is known for its anti-hyperglycemic and antioxidant properties [26], enhancement of insulin sensitivity, and reduction of oxidative stress, as recently reported by Cao and Su. It stimulates glucose uptake by activating the AMP-activated protein kinase (AMPK) pathway, promoting better BW management in diabetic conditions [27]. Apart from the significant restoration of BW, other important activities of *BA* include reduced gluconeogenesis and anti-inflammatory effects, which have also been reported by Zhang et al. and Ehteshamfar et al., in 2018 and 2020, respectively [28,29]. In our study, *BA* administration at different dosages resulted in significant attenuation of BW loss in diabetic rats, which can be linked to its ability to improve metabolic functions and reduce muscle wasting and adipose tissue loss.

WS

Ashwagandha is a well-known traditional Indian herb with adaptogenic properties that provide various health benefits, which are gradually being documented. Some of the documented properties include endocrine, cardiopulmonary, central nervous system, and sexual behavior effects, all without any reported toxicity. Several studies report properties such as immunomodulatory, anti-inflammatory, antistress, memory-enhancing, antiparkinsonian, hypolipidemic, antibacterial, cardiovascular, antioxidant, and antitumor effects, as reviewed and reported by John [30]. Ashwagandha reduces stress induced by hyperglycemia, modulates cortisol levels, as reported by Bhattacharya and Muruganandam [31], improves overall metabolism, and counters stress-induced catabolism, aiding in better maintenance of BW [32]. Established studies by Bonilla et al. [33] and Wankhede et al. [34] have shown that *WS* promotes muscle mass retention and improves body composition by increasing anabolic activity and reducing catabolic stress hormones.

Similar animal experiments conducted by Ko et al. reported significant increases in muscle mass and strength [35]. In our study, the administration of *WS* in diabetic rats showed a reduction in weight loss, which may be due to its muscle-preserving effects and reduction in oxidative stress.

PHC

The combination of *BA* and *WS* was hypothesized to have a synergistic effect due to their complementary mechanisms of action, such as enhanced glycemic control, improved insulin sensitivity, reduced inflammation, synergistic antioxidant effects, and adaptogenic properties [36,37]. The treatment with PHC in our study resulted in the most significant attenuation of BW loss among the groups, indicating a potential synergistic effect of *BA* and *WS* in managing BW in STZ-NIC-induced T2DM in Wistar rats.

Strengths and limitations

Strengths

Clear research question: The study provides a focused investigation into the effects of *BA*, *WS*, and their combination on BW in T2DM Wistar rats. This focus is particularly relevant, given the significant impact of diabetes-induced muscle wasting and weight loss.

Well-established animal model: The study utilized the STZ-NIC-induced T2DM Wistar rat model, which is a well-established and widely accepted model for mimicking human T2DM, including its metabolic dysfunctions. This enhances the relevance and potential applicability of the findings to human diabetes management.

Detailed methodology: The materials, animal handling, drug administration, and statistical analysis are clearly described.

Dose-response analysis: The study included multiple doses of *BA* and *WS*, allowing for a detailed analysis of the dose-response relationship. This approach helps to identify the most effective doses for BW management and provides a basis for future therapeutic recommendations.

Positive findings: The study demonstrates that the highest dose of *BA* (1000 mg/kg) and *WS* (1000 mg/kg) improves BW in diabetic rats.

Exploration of combination therapy: The study investigated the effects of combining *BA* and *WS* in a 1:1 ratio, hypothesizing potential synergistic effects. The observed improvement in BW with combination therapy suggests enhanced therapeutic potential, which is a key strength of the study.

Limitations

Lack of additional outcome measures: While the study focused on BW, it did not include other important metabolic parameters, such as blood glucose levels, insulin sensitivity, or lipid profiles, within the same publication. Although these data were published elsewhere, their inclusion could have provided a more comprehensive understanding of the metabolic effects of *BA*, *WS*, and their combination.

No visual documentation: The study did not capture visual images of the rats, which could have provided additional qualitative data on the physical condition of the animals, and enhanced the presentation of the findings.

Mechanistic insights: The study primarily focused on the outcomes, rather than the underlying mechanisms by which *BA* and *WS* improve BW. While some mechanistic pathways were discussed, detailed molecular analyses were not conducted within this study.

Limited exploration of synergistic effects: Although the combination therapy was explored, the study only tested a single ratio (1:1) of *BA* and *WS*. Testing additional ratios could have provided deeper insights into the optimal combination and interaction effects between the two herbs.

Single focus: The exclusive focus on BW as the primary outcome measure, without concurrent analysis of muscle mass or fat composition, limits the ability to fully understand the nature of the weight changes observed, such as whether the weight gain was due to muscle mass preservation or fat accumulation.

## Conclusions

This study highlights the significant potential of *BA*, *WS*, and PHC in managing BW in STZ-NIC-induced T2DM Wistar rats. Particularly at higher doses, these herbs suggest their potential as effective complementary adjunct therapies in the management of diabetes-induced weight loss. The findings indicate that *BA*, *WS*, and PHC therapy may offer superior benefits by leveraging the complementary mechanisms of both plants. Further research and clinical trials are required to explore their full therapeutic potential and mechanisms of action in humans, aiming to elucidate the precise biochemical pathways through which these herbs exert their beneficial effects and evaluate the long-term safety and efficacy of such combination therapies in diabetes management.

## References

[1] Khardori R (2024). Type 2 Diabetes Mellitus, Practice Essentials, Background, Pathophysiology. https://emedicine.medscape.com/article/117853-treatment.

[2] Goyal R, Singhal M, Jialal I (2024). Type 2 diabetes. StatPearls [Internet].

[3] Liu Z, Guo Y, Zheng C (2024). Type 2 diabetes mellitus related sarcopenia: a type of muscle loss distinct from sarcopenia and disuse muscle atrophy. Front Endocrinol (Lausanne).

[4] Chobot A, Górowska-Kowolik K, Sokołowska M, Jarosz-Chobot P (2018). Obesity and diabetes-not only a simple link between two epidemics. Diabetes Metab Res Rev.

[5] Tiwari DD, Thorat VM, Pakale PV, Patil SJ (2024). Study of antidiabetic properties of Berberis asiatica and Withania somnifera in streptozotocin-nicotinamide-induced type II diabetes mellitus in Wistar rats. Cureus.

[6] Yan LJ (2022). The nicotinamide/streptozotocin rodent model of type 2 diabetes: renal pathophysiology and redox imbalance features. Biomolecules.

[7] Liu H, Tu M, Yin Z, Zhang D, Ma J, He F (2024). Unraveling the complexity of polycystic ovary syndrome with animal models. J Genet Genomics.

[8] Cruz PL, Moraes-Silva IC, Ribeiro AA (2021). Nicotinamide attenuates streptozotocin-induced diabetes complications and increases survival rate in rats: role of autonomic nervous system. BMC Endocr Disord.

[9] Sharma S, Chaitanya MVNL, Sharma S (2024). The medicinal plant Berberis aristata and its endophytes for pharmacological applications: Current research and future challenges. J Appl Biol Sci.

[10] Bhat JA, Kumar M, Negi AK, Todaria NP, Malik ZA, Nazir A (2020). Species diversity of woody vegetation along altitudinal gradient of the Western Himalayas. Glob Ecol Conserv.

[11] Jahan F, Alvi SS, Mohammad HI (2022). Berberis aristata and its secondary metabolites: insights into nutraceutical and therapeutical applications, pharmacological research. Mod Chin Med.

[12] Neag MA, Mocan A, Echeverría J, Pop RM, Bocsan CI, Crişan G, Buzoianu AD (2018). Berberine: botanical occurrence, traditional uses, extraction methods, and relevance in cardiovascular, metabolic, hepatic, and renal disorders. Front Pharmacol.

[13] Palani D, Mahaboobkhan R. (2019). Herbal formulations and their bioactive components as dietary supplements for treating rheumatoid arthritis. Bioactive Food as Dietary Interventions for Arthritis and Related Inflammatory Diseases (Second Edition).

[14] Singh N, Bhalla M, de Jager P, Gilca M (2011). An overview on ashwagandha: a rasayana (rejuvenator) of ayurveda. Afr J Tradit Complement Altern Med.

[15] Widodo N, Kaur K, Shrestha BG, Takagi Y, Ishii T, Wadhwa R, Kaul SC (2010). Selective killing of cancer cells by ashwagandha leaf extract and its component withanone involves ROS signaling. PLoS One.

[16] Mirjalili MH, Moyano E, Bonfill M, Cusido RM, Palazón J (2009). Steroidal lactones from Withania somnifera, an ancient plant for novel medicine. Molecules.

[17] Mishra LC, Singh BB, Dagenais S (2000). Scientific basis for the therapeutic use of Withania somnifera (ashwagandha): a review. Altern Med Rev.

[18] Kulkarni SK, Dhir A (2008). Withania somnifera: an Indian ginseng. Prog Neuropsychopharmacol Biol Psychiatry.

[19] Bhutada P, Mundhada Y, Bansod K, Tawari S, Patil S, Dixit P, Mundhada D (2011). Protection of cholinergic and antioxidant system contributes to the effect of berberine ameliorating memory dysfunction in rat model of streptozotocin-induced diabetes. Behav Brain Res.

[20] Kartal D, Ozok N (2021). Anti-hyperglycemic effect of <i>Vicia ervilia</i> (L.) Willd (Fabaceae) seed extract and its effect on lipid profile, and hepatic and renal biomarkers in streptozotocin-induced diabetic rats. Trop J Pharm Res.

[21] Saggam A, Tillu G, Dixit S, Chavan-Gautam P, Borse S, Joshi K, Patwardhan B (2020). Withania somnifera (L.) Dunal: a potential therapeutic adjuvant in cancer. J Ethnopharmacol.

[22] (2024). OECD guidelines for the testing of chemicals, section 2. https://doi.org/10.1787/9789264242258-en.

[23] Rakha A, Ramzan Z, Umar N (2023). The role of ashwagandha in metabolic syndrome: a review of traditional knowledge and recent research findings. J Biol Regul Homeost Agents.

[24] Rani R, Dahiya S, Dhingra D, Dilbaghi N, Kaushik A, Kim KH, Kumar S (2019). Antidiabetic activity enhancement in streptozotocin + nicotinamide-induced diabetic rats through combinational polymeric nanoformulation. Int J Nanomedicine.

[25] Bays HE (2023). Why does type 2 diabetes mellitus impair weight reduction in patients with obesity? A review. Obes Pillars.

[26] Lee YS, Kim WS, Kim KH (2006). Berberine, a natural plant product, activates AMP-activated protein kinase with beneficial metabolic effects in diabetic and insulin-resistant states. Diabetes.

[27] Cao C, Su M (2019). Effects of berberine on glucose-lipid metabolism, inflammatory factors and insulin resistance in patients with metabolic syndrome. Exp Ther Med.

[28] Zhang B, Pan Y, Xu L (2018). Berberine promotes glucose uptake and inhibits gluconeogenesis by inhibiting deacetylase SIRT3. Endocrine.

[29] Ehteshamfar SM, Akhbari M, Afshari JT (2020). Anti-inflammatory and immune-modulatory impacts of berberine on activation of autoreactive T cells in autoimmune inflammation. J Cell Mol Med.

[30] John J (2014). Therapeutic potential of Withania somnifera: a report on phyto-pharmacological properties. Int J Pharm Sci Res.

[31] Bhattacharya SK, Muruganandam AV (2003). Adaptogenic activity of withania somnifera: an experimental study using a rat model of chronic stress. Pharmacol Biochem Behav.

[32] Panda S, Kar A (1999). Withania somnifera and Bauhinia purpurea in the regulation of circulating thyroid hormone concentrations in female mice. J Ethnopharmacol.

[33] Bonilla DA, Moreno Y, Gho C, Petro JL, Odriozola-Martínez A, Kreider RB (2021). Effects of ashwagandha (Withania somnifera) on physical performance: systematic review and bayesian meta-analysis. J Funct Morphol Kinesiol.

[34] Wankhede S, Langade D, Joshi K, Sinha SR, Bhattacharyya S (2015). Examining the effect of Withania somnifera supplementation on muscle strength and recovery: a randomized controlled trial. J Int Soc Sports Nutr.

[35] Ko JS, Chang BY, Choi YJ (2024). Ashwagandha ethanol extract attenuates sarcopenia-related muscle atrophy in aged mice. Nutrients.

[36] Sonam KS, Guleria S (2017). Synergistic antioxidant activity of natural products. Ann Pharmacol Pharm.

[37] Kuritzky L, Samraj GP (2011). Enhanced glycemic control with combination therapy for type 2 diabetes in primary care. Diabetes Ther.

